# The Influence of Ultra-Wideband Anchor Placement on Localization Accuracy

**DOI:** 10.3390/s25165115

**Published:** 2025-08-18

**Authors:** Luka Kramarić, Mario Muštra, Tomislav Radišić

**Affiliations:** Faculty of Transport and Traffic Sciences, University of Zagreb, HR-10000 Zagreb, Croatia; lkramaric@fpz.unizg.hr (L.K.); tradisic@fpz.unizg.hr (T.R.)

**Keywords:** UAV, UWB, localization, anchor, time of arrival, two-way ranging, laser altimeter

## Abstract

Localization of Unmanned Aerial Vehicles (UAVs) in spaces with a limited availability of Global Navigation Satellite System signals presents a challenge, and one possible solution is the usage of Ultra-Wideband (UWB) transceivers as an aid in the localization process. This paper examines the influence of placing the UWB anchors on the UAVs’ localization accuracy in indoor spaces. Different testing scenarios, with variations in the number of anchors and their relative position towards the UAV, were created. Results show that the anchor placement plays an important role and is a significant factor in achieving accurate positioning of UAVs. The error for different testing configurations was shown through the RMSE for each axis, backed up by the standard deviation. The increase in the number of UWB anchors with the combined use of an additional laser ranging sensor for altitude measurement provided the best result. The RMSE was less than 18 cm in each axis of a 3D coordinate system with the standard deviation of up to 4.41 cm. For the testing scenarios that included the usage of a laser altimeter, the RMSE for the *z*-axis dropped below 1 cm, with the standard deviation of under 0.3 cm.

## 1. Introduction

The recent development and maturing of Unmanned Aerial Vehicle (UAV) technology opened different fields of research related to their navigation, both in outdoor and indoor environments. Depending on the actual use cases and requirements, UAVs are equipped with various navigation systems, providing them with capabilities of global and local positioning. In the outdoor environments, with the moderate requested accuracy, usage of the Global Navigation Satellite Systems (GNSSs) proves sufficient, with the expected accuracy of up to a couple of meters. The recent development of imaging devices, including visible light (RGB) and multispectral cameras and LiDARs, paved the way for the usage of rather cheap equipment for photogrammetric tasks [1]. The standard precision positioning GNSS signal is not sufficiently accurate for most of the tasks UAVs are meant to perform [2] and the Real-Time Kinematic (RTK) is usually the solution for achieving the centimeter-level positioning accuracy [3]. RTK proves to be accurate enough for mapping of objects and terrain or agricultural usage, but it requires a base station or access to a GNSS reference station in real-time. Even though this setup is rather easy to achieve, it is not an ad-hoc solution, and the usable results are possible only in outdoor environments. Knowing the exact position is necessary in single- or multi-UAV systems, but the relative position in multi-UAV systems is not guaranteed with the usage of satellite-based positioning alone. To cope with the operation of autonomous swarms and for indoor navigation of individual UAVs, or swarms of UAVs, is the main reason why the relative localization is being investigated by many researchers [4].

Among the relative localization methods, Ultra-Wideband (UWB) localization technology provides a solution to achieve a centimeter (or decimeter) level accuracy, which should enable the usage of UAVs in indoor environments and provide the additional capability to improve the relative localization of individual UAVs in swarms outdoors. UWB is basically a wireless communication technology operating in a wide frequency spectrum, from 3.1 GHz to 10.6 GHz [5]. It is used to transmit and receive signals between the UAV and the pre-built infrastructure or between two or more UAVs. UWB technology relies on the usage of extremely short pulses, typically in the order of nanoseconds, requiring a wide frequency range. There are two typical approaches in UWB-based localization: the time of flight (ToF) and the time difference of arrival (TDoA). These approaches are sometimes backed up by the angle of arrival (AoA), which can help in reducing the error by using the information about the angle from which the signal arrives.

In addition to the unavailability of the GNSS signal in indoor environments, the structure of the space, and the material from which it is built, play an important role, because they can lead to undesired effects of multipath signal propagation, where unwanted reflections from walls and objects lead to incorrect measurements on which the localization accuracy relies [6,7]. In this paper, we will show the results of the influence of the surroundings and relative position between the UWB anchors and a UAV in different configurations and provide guidelines for choosing the optimal setup regarding the potential use case. The real-world usage of a system is usually somewhat compromised by the possibilities to place the localization equipment at optimal places and those compromises could lead to an increase in the dilution of precision (DOP), which we will show through intentionally falsely chosen locations of anchors. Depending on the possibility of placing the anchors and because a UAV can move in three dimensions, the vertical component may have a larger error, especially when anchors are in the same horizontal plane. To overcome that problem, we will provide a possible solution by introducing augmentation using an additional laser-based altitude sensor and compare the localization accuracy with and without it.

The rest of the paper is structured as follows: Section 2 brings a brief literature overview covering the research activities in the field of UWB-based localization. Section 3 provides a description of the equipment used, its limitations, and achievable accuracy. Section 4 describes the methodology and shows the experimental setup used throughout the experiments. Section 5 shows the results of measurements and provides a discussion about the achievements through different configurations of anchors and their relative positions towards the UAV. Finally, Section 6 brings conclusive remarks and guidelines for the future work.

## 2. The Recent Research in UWB-Based Localization

UWB-based localization, especially in indoor environments, has been considered in many studies in recent years. Although it is a well-known principle, there are many directions in which researchers have pointed their attention. One of the biggest challenges of the UWB technology used in positioning is the influence of the mutual position between the fixed points (anchors) and targets, which usually move, either on the ground or fly in three dimensions. Cho et al. [8] were trying to determine the optimal location of UWB tags on a construction site to obtain the best localization accuracy of a mobile terminal. The mobile terminal could measure the actual distance between each tag and to determine its location, the author used trilateration, which was proprietarily developed. Depending on the location of tags, the value of positional DOP (PDOP) varied from 1.50 to 3.39 and even to infinity, in the case of coplanar location of tags, which increased the vertical DOP so much that the system could not provide 3D position accurately. They achieved an accuracy of around 35 cm in 3D positioning, using the proposed method. Monica and Ferrari [9] were trying to locate the target in an industrial environment with automated ground vehicles using the TDoA-based method. In their simulation, the vehicle was able to move only on the ground, which reduced the complexity of the network of anchors, and the favorable location was chosen to be above the ground vehicle on the ceiling with as large as possible angle between the anchors and the vehicle, which significantly reduced the vertical DOP. In addition to the open spaces, where the line of sight (LoS) is present, many use-cases include non-LoS environments (NLoSs), where there is a complete or partial occlusion between the anchors and the target. These scenarios were investigated by Pan et al. [10] and Albaidhani et al. [11]. In both cases, the localization accuracy was significantly lower and not acceptable for the precise usage of UAVs indoors. To improve the 3D accuracy, Pan et al. [12] proposed coplanar placement of UWB anchors and proved that the overall error is significantly reduced with the optimal placement of UWB anchors. Cerro et al. [13] compared the performance of different available hardware for UWB-based positioning: Decawave, BeSpoon, and Ubisense. The tests were carried out for different numbers and positions of anchors, and in static and dynamic conditions. The authors set the error threshold to be 60 cm and provided a comparison of the device’s performance for 4 to 12 anchors in two configurations. Their results show that the Decawave hardware provides a slightly better and more consistent performance, which corresponds well to the results Ruiz and Granja [14]. The obtainable error was within 60 cm for nine or more anchors and for all of the tested equipment. Ferrigno et al. [15] also dealt with the anchor placement and robustness of the UWB-based localization using the Decawave hardware and, through the experiment, obtained errors from 22 cm to 72 cm. It is important to note that the authors used only three anchors, which somewhat reduced the localization accuracy of the system. Depending on the characteristics of the environment where the UWB-based localization is performed, Chen et al. [16] proposed system for indoor positioning with optimal anchor location, and showed that the influence of obstacles plays a significant role in positioning accuracy, with the suitable performance achieved only in LoS environments. Another recently proposed method for choosing an optimal anchor position was presented by Pan et al. [17] who used a heuristic differential evolution algorithm for searching for an optimal anchor placement that is based on minimizing the Cramer–Rao lower bound and predicting the ranging error. Zhao et al. [18] proposed a block coordinate-wise minimization algorithm to search for the optimal anchor configuration by reducing the average RMSE. They carried out experiments in different environments, with and without obstacles, that are mostly made of electrically neutral material and concluded that it is necessary to have 12 or more anchors to provide a localization accuracy of around 5 cm on a narrow path among obstacles. During the last decade, many papers dealing with the usage of UWB for localization purposes in industrial environments were published [19,20,21,22,23,24,25], making this technology highly interesting for automation and motion control. In addition to UWB-based localization, there were some attempts to use different communication technologies for indoor localization, e.g., wireless local-area networks with access points acting as anchors [26]. Because of the technological limitations, this approach, no matter which distribution of access points the authors used, could not provide an accuracy in localization better than couple of meters.

Autonomous landing is one of the special cases that includes the usage of the UWB technology, where Nguyen et al. [27] used the UWB technology to aid visual-based docking in the approach phase, which lasts until the visual sensors recognize the markers on the landing pad. Miranda et al. [28] combined the usage of the UWB technology, IMU, and GPS in landing a delivery UAV in a designated area.

Operating swarms of UAVs is rather challenging if the relative localization is performed through the exchange of relative distances between individual UAVs. Guo et al. [29] proposed a relative localization achieved through a distance-based method for UAV swarm formation control and successfully managed to maintain the equilateral triangle formation in a leader–follower technique, in which the leader UAV is manually controlled and moves freely, while other UAVs are following it. The mentioned-before AoA is not something that can be estimated from the UWB signal and requires the use of a direction-sensing antenna, which has completely different properties from the antennas on UWB tags. The approach utilizing an antenna array for measuring both the distance and the angle between two UAVs was presented by Gu et al. [30]. The authors reported the accuracy of around 23 cm at a speed of 0.64 m/s.

Simultaneous localization and mapping (SLAM) is another research field where UWB localization technology is used. One of the studies dealing with the choice of the correct equipment for SLAM is from Gerwen et al. [31]. They analyzed which sensors are the best suited to which UAVs, respective to their size, and compared the accuracy obtained by using a different number of anchors. An interesting outcome of this study is the comparison of accuracy after aiding UWB localization technology with a sonar. The authors obtained an average positioning error of 67 cm in 3D and after adding an inexpensive altitude sonar, the average error dropped to 10.71 cm. Although this reduction in the positioning error is significant, it is still rather high for many use cases. A movable localization system relying on UWB, an attitude and heading reference system, and a barometer for altitude augmentation was proposed by Moon and Yoon [32], where they have shown how the sole usage of a UWB-based localization system is not sufficient on its own. As mentioned before, the accurate estimation of altitude (*z*-axis) is usually a more difficult task due to the geometry of the anchors in the majority of indoor localization methods and one of the approaches is to include additional information provided by different sensors, as did Si et al. [33] and Yang et al. [34] with the combination of a barometer and UWB, similar to the work of Zhang et al. [35] for swarms of UAVs.

## 3. UWB Equipment Used in Experiments

**Figure 1:**
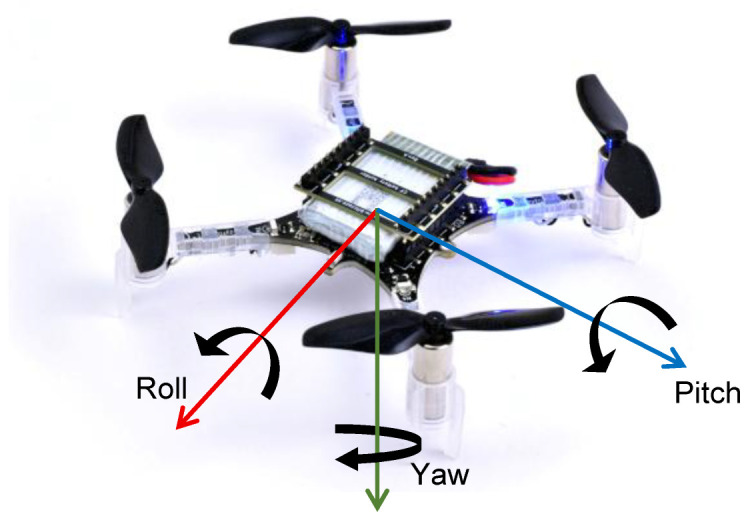
The Crazyflie 2.1+ UAV from Bitcraze AB that was used in the experiments with roll, pitch, and yaw angles.

To test the various scenarios of placement of UWB anchors and influence on the localization accuracy of the UAV, we have used the ecosystem from Bitcraze AB, Malmö, Sweden, consisting of the UAV (Crazyflie) equipped with STM32F405 microcontroller unit (MCU) and nRF51822 radio and power management MCU, as shown in Figure 1.

**Figure 2:**
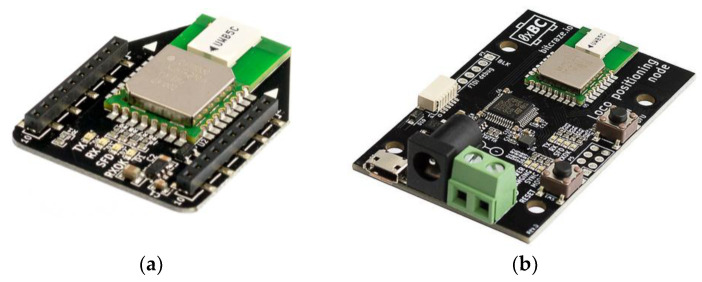
The hardware used in the experiments: (**a**) Loco Positioning expansion deck used on the UAV; (**b**) Loco Positioning node used for anchors in various configurations.

The UWB hardware is slightly different in the case of the rover (Loco Positioning expansion deck) and the anchor (Loco Positioning node), as shown in Figure 2a and Figure 2b, respectively.

In terms of UWB transceivers, both devices use the same Decawave DWM1000 module compatible with the IEEE 802.15.4 UWB specification. The operating bandwidth is from 3.2 GHz to 7 GHz with a channel bandwidth of 500 MHz. In the experiments we used the channel 2, with the center frequency of 3993.6 MHz and the bandwidth of 499.2 MHz. The pulse repetition frequency was set to 64 MHz, which creates the data rate of 6.81 Mbps [36]. The manufacturer provided the calibration for these settings and the double-sided two way-ranging (DS-TWR) at 8.1 m [37]. The ranging rate is 500 Hz, which is distributed among the number of anchors used in each setup. This means that the ranging rate per anchor can be calculated using the following expression:

(1) $$Rfa≲\frac{Rf}{Na},$$

where *Rfa* is the ranging frequency per anchor, *Rf* is the achievable ranging frequency of the expansion deck, and *Na* is the number of used anchors. The anchors are built around the STM32F072 MCU and the configuration can work with up to eight anchors simultaneously. It needs to be noted that it is required to have four or more anchors to achieve a good 3D-localization, meaning that variation in the number of anchors using this hardware is rather limited. The manufacturer claims that both devices can achieve a ranging accuracy of around ±10 cm, which corresponds well to the claimed performance of the DWM1000 module. The used transceivers operate at 3.3 V and the positioning frequency was set at 25 Hz. This setup enabled accurate positioning at distances of up to 10 m.

Height estimation proves to be more challenging than horizontal positioning and for that reason, we decided to use an additional sensor to estimate the height of the UAV from the floor and merge its readings with the UWB-based localization. The used sensor (Z-ranger deck v2, from Bitcraze AB) is based on the VL53L1x ToF sensor, capable of measuring distances up to 4 m with an average error within a few millimeters.

## 4. Methodology and the Experimental Setup

**Figure 3:**
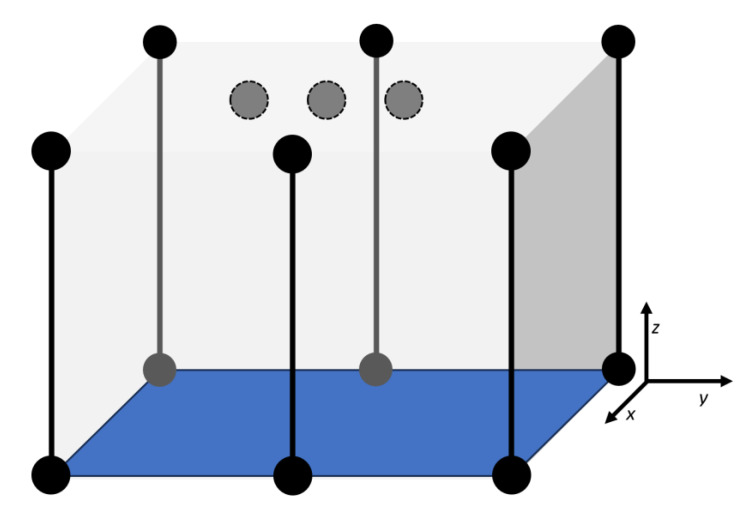
The volume inside which the localization accuracy was measured with the used coordinate system. Circles represent UWB anchors, gray dashed circles show possible locations of overhead UWB anchors, and vertical lines represent columns along which UWB anchors can change their altitude.

In the previous section, we mentioned that 3D positioning requires at least four anchors and the maximum supported number of anchors is eight. This research aims to create guidelines for the placement of anchors to achieve maximum positioning accuracy and observe the influence of indoor environments. Indoor spaces are often height limited, so we will place anchors and allow movement of the UAV inside and in proximity of the volume defined by the anchor positions, shown in Figure 3. In actual applications, the anchor positions are usually in the bottom corners of the room where positioning takes place and some authors suggested placement of anchors above the UAV, in the center of the room, or the top corners. These approaches make sense in terms of reducing the DOP factor, which influences the final poisoning accuracy and is defined by:

(2) $$\Delta =PDOP\cdot \delta_{R},$$

where *Δ* is the final positioning error in meters, *PDOP* is the positional dilution of precision, which consists of the horizontal and vertical dilution of precision, and *δ_R_* is the error in ranging caused by the sensor. Placing the target inside the volume defined by the position of the anchors will reduce the (P)DOP, as shown in [38]. (P)DOP was calculated at several points of interest. The first step in the calculation is building the geometry matrix **H** for each position of interest ($\hat{x},\;\hat{y},\hat{z}$):

(3) $$H=\left[\begin{array}{ccc} \frac{x_{1}-\hat{x}}{\rho_{1}} & \frac{y_{1}-\hat{y}}{\rho_{1}} & \frac{z_{1}-\hat{z}}{\rho_{1}}\\ \frac{x_{2}-\hat{x}}{\rho_{2}} & \frac{y_{2}-\hat{y}}{\rho_{2}} & \frac{z_{2}-\hat{z}}{\rho_{2}}\\ \vdots & \vdots & \vdots \\ \frac{x_{N}-\hat{x}}{\rho_{N}} & \frac{y_{N}-\hat{y}}{\rho_{N}} & \frac{z_{N}-\hat{z}}{\rho_{N}} \end{array}\right],$$

where ($x_{i},\;y_{i},\;z_{i}\;$) are anchor positions, ($\hat{x},\;\hat{y},\;\hat{z}$) is the position of interest, and $\rho_{i}$ is the distance to anchor *i.* The next step is to calculate the covariance matrix **Q** as:

(4) $$Q={\left(H^{T}H\right)}^{-1}.$$

The final step is calculating the *PDOP* value as:

(5) $$PDOP=\sqrt{Q_{xx}+Q_{yy}+Q_{zz}},$$

where *Q_xx_*, *Q_yy_*, and *Q_zz_* are the diagonal elements of the matrix **Q**.

We aimed to evaluate the accuracy and stability of UWB-based localization in an indoor environment for a static UAV using different anchor configurations, with four to six anchors used simultaneously. The UAV was placed at fixed positions in space so that its position is static and always known, with the roll and pitch angles set at 0°, which should correspond well with the expected orientation at low speeds and in hovering. The main objective was to analyze how the anchor geometry and sensor fusion, specifically the inclusion of a laser altimeter with the millimeter-level accuracy, influence the positional accuracy along the *x*, *y*, and *z* axes. Key performance indicators include Root Mean Square Error (RMSE), standard deviation (STDEV), and average position (AVG) in each axis. The RMSE was calculated as:

(6) $$RMSE=\sqrt{\frac{\sum_{i=1}^{N}{\left(d_{i}-d\right)}^{2}}{N}}$$

where *N* is the number of measurements, *d_i_* is the *x* or *y* component of *i*-th measurement, and *d* is the actual position of the UAV. The standard deviation was calculated using:

(7) $$STDEV=\sqrt{\frac{\sum_{i=1}^{N}{\left(d_{i}-\hat{d}\right)}^{2}}{N}}$$

where $\hat{d}$ is the average (AVG) of the measurements.

**Figure 4:**
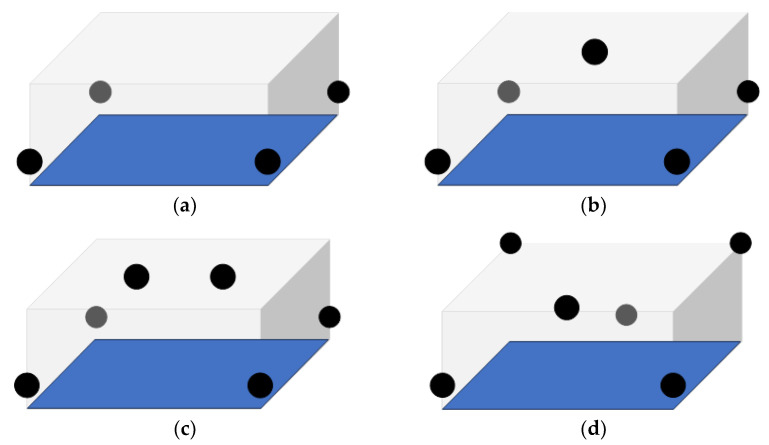
(**a**) Configuration 1, where all the anchors are placed in the same horizontal plane 40 cm above ground covering the area of 4 × 3 m; (**b**) Configuration 2, similar to the Configuration 1 with the additional anchor in the center at the height of 2 m; (**c**) Configuration 3 with two anchors equally spaced at the height of 2 m; (**d**) Configuration 4 with three anchors in the form of a triangle at one plane and three symmetrical upside-down on the opposite plane.

The UWB localization method used in the experiment is based on ToF measurements between a tag (attached to the UAV) and multiple fixed anchors. The DS-TWR was used to precisely calculate the ToF. The position of the tag is calculated using trilateration, a mathematical method that finds a point in space based on its distances from known reference points, which can be written as:

(8) $${\left(x-x_{i}\right)}^{2}+{\left(y-y_{i}\right)}^{2}+{\left(z-z_{i}\right)}^{2}={d_{i}}^{2};\;i\in \left[1,\;n\right],$$

where (*x_i_*, *y_i_*, *z_i_*) are coordinates of the *i*-th anchor in the Euclidean space, (*x*, *y*, *z*) are coordinates of the tag (unknown), *d_i_* is the distance between each anchor and the tag, and *n* is the number of anchors, which varies from four to six in our case. Solving this system requires at least four non-coplanar anchors if there is no additional information of the dimension perpendicular to the plane defined by the anchors. Using more anchors improves redundancy and mitigates errors caused by signal noise, non-line-of-sight (NLoS) conditions, or poor geometry (high *DOP* factor). To evaluate how the spatial distribution of anchors affects UWB localization accuracy, four unique configurations were tested. Each configuration increased the spatial diversity and number of anchors progressively, allowing a structured comparison of their impact on position estimation. Table 1 presents the details of the configurations used in experiments, while the configurations used (1–4) are shown in Figure 4a–d, respectively. The dimensions of the room used for experiments are rather limited, which did not introduce possible problems of signal attenuation, and the anchor positions could be chosen to have a good spatial distribution, which might not be the case in larger or oddly shaped environments. The anchor positions and distance measurements were made using a laser measuring device with the accuracy of ±0.015 cm.

The UAV was hanging from the support mounted on a wooden pole using a non-elastic thread, ensuring a fixed and stable position in 3D space throughout all measurements. This eliminated dynamic motion effects and allowed for the evaluation of system accuracy in a controlled and repeatable environment. The known static position of the UAV served as the reference for performance evaluation. Four distinct anchor configurations were designed to progressively increase the geometric diversity and redundancy of the localization system. Each configuration was tested under the same environmental conditions to ensure comparability, and additionally, each configuration was tested with and without the inclusion of the laser-based altimeter. The altimeter was integrated to provide altitude correction and improve *z*-axis stability, especially important in coplanar or nearly coplanar anchor setups. This laser-based solution is possible to be used only in the indoor environments due to the sunlight interference and for an outdoor use an RTK-GPS would be an advisable solution, although the expected positioning error would increase.

## 5. Measurement Results and Discussion

The objective of the experiments was to evaluate the accuracy and performance of the static UAV’s position estimation in different UWB anchor configurations and to observe the influence of a relative distance to different anchors on the accuracy of position estimation. The measurements were performed at three fixed positions in the *xy*-plane and at three different heights. The location estimation was performed using the Bitcraze Loco Positioning System with DS-TWR. In this mode, no synchronization between anchors is required, as the tag actively ranges with each anchor individually and measures the distance using the round-trip time. Measurements were logged via the Crazyflie Python-based client over the radio through Crazy RealTime protocol at 25 Hz. The raw distance data and estimated positions were exported as CSV files for further analysis. All the visualizations and post processing were performed in Python 3.10.0rc2 using Matplotlib 3.10.3.

### 5.1. Measurement Results

**Figure 5:**
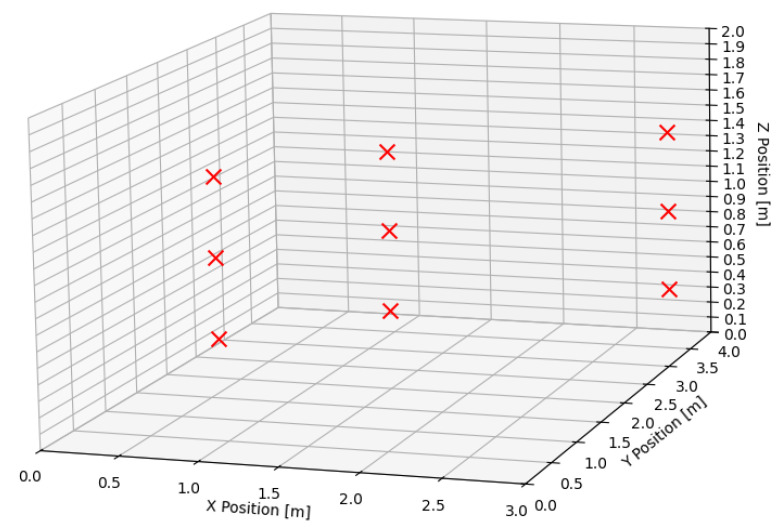
The positions at which the UAV was fixed are depicted with red Xs. These positions were selected to provide a relatively low PDOP and to test the localization accuracy at different heights.

A total of four UWB anchor configurations were tested to evaluate their effect on positioning accuracy. Each configuration was tested under the same conditions in a 3 × 4 × 2-m indoor space, as described in the previous section. Figure 5 shows the positions at which the UAV was fixed to perform measurements. Each pair of (*x*, *y*) coordinates was measured at three different heights and each point was averaged over 500 samples with and without the ToF-based laser height sensor. To evaluate the overall accuracy, the RMSE and the STDEV in the *x* and *y* axis were calculated for each configuration, with results aggregated across all three test points and all height levels. Table 2 presents the average results and serves to provide the expected error in positioning inside a similarly sized volume. It can be seen that in the case of coplanarly positioned anchors, the RMSE and the standard deviation of the *z*-axis position estimation become unacceptably high.

**Figure 6:**
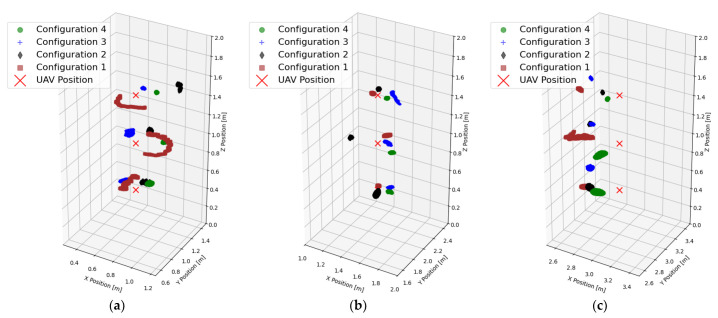
3D scatter plots for three different horizontal locations and three different heights. (**a**) Point 1 corresponds to the location of (0.75, 1.0) m. (**b**) Point 2 corresponds to the location of (1.5, 2.0) m. (**c**) Point 3 corresponds to the location of (3.0, 3.0) m.

Another important factor that significantly influences the final achieved accuracy of any localization system is the PDOP. Depending on the measured unit and nature of the system meant to provide accurate position, it can be expected that the PDOP is usually smaller towards the center of the volume stretched by anchors of a non-autonomous localization system. Table 3 presents calculated PDOPs for the Configuration 1, where P1 corresponds to the location of (0.75, 1.0) m, P2 corresponds to the location of (1.5, 2.0) m, and P3 corresponds to the location of (3.0, 3.0) m, H1 corresponds to the height of 0.5 m, H2 corresponds to the height of 1.0 m, and H3 corresponds to the height of 1.5 m. Depending on the chosen x and y coordinates, Figure 6a–c show 3D scatter plots of estimated UAV locations using four anchor configurations and present the influence of configuration and PDOP on the expected accuracy of localization.

**Figure 7:**
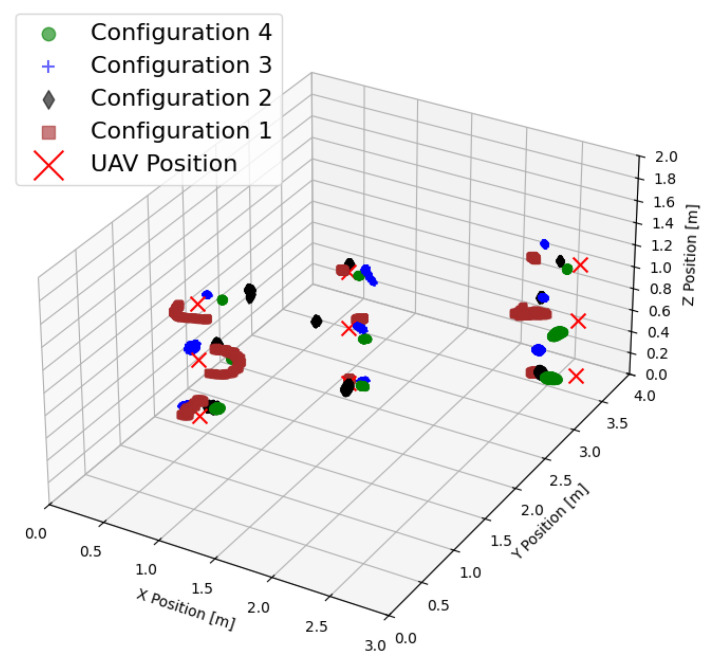
Combined results of the position estimation for all configurations and all points at which the measurements took place.

The UWB-based localization struggles in Configuration 1, where anchors are placed coplanarly, but shows a sufficient accuracy in other configurations. However, adding a lightweight and inexpensive laser height sensor eliminated the *z*-axis error almost completely. Across all the configurations, the RMSE of *x* and *y* ranged between 11 cm and 18 cm, with Configuration 4 offering the most accurate and consistent results. From Table 3, it is obvious that choosing a coplanar anchor setup leads to large values of the PDOP, where everything above 2, and especially 10 or even more, is unacceptable and leads to significant positioning errors. The combined results for all four configurations at nine different locations are shown in Figure 7.

### 5.2. Discussion

During the measurements and through the results of measurements, shown in the previous subsection, we observed a consistent spatial bias, likely related to the anchor placement geometry or a potential miscalibration of the system. This is somewhat expected in every real-world application, because it is rather difficult to set up an anchor system without any absolute positional error, and there is a question whether that is always necessary, since the UWB-based localization relies on the relative distance between transceivers. The system used in the experiments is built around the DWM1000 module and the manufacturer states that the ranging bias depends on multiple factors: the received signal strength, the pulse repetition frequency, and the clock drift [37]. These factors are not trivial to compensate for and for them to be partially or completely eliminated, it would be necessary to perform the initial calibration of each module before each measurement. Estimated positions tend to cluster in a tight cloud with a small offset from the real location of the UAV, as shown in Figure 6, which is good for maintaining a relative position. This bias appears to be stable across all tested configurations, implying it is a systematic bias rather than a random one. Because the bias is repeatable, it may be possible to correct it through simple calibration or error modelling and provide even more accurate position estimation. A developed system with a similar architecture could benefit more from an initial bias compensation than from optimizing anchor configurations, if Configuration 1 is to be taken out of consideration, because of its lack of variation in the *z*-axis. Even though the indoor space was controlled and cleared of almost all metallic objects, it inevitably introduced multipath reflections. This has likely contributed to some noise in the position estimates, which further increased the RMSE. Using a UWB-based positioning in irregularly shaped environments with electromagnetically reflexive obstacles would require a better definition of the space and the information about the position of each obstacle which would have to be shared with each UAV and treated as a no-fly zone with an appropriate safe margin around it.

Diversity in height of anchors from the ground plane (floor), generally, provides better results, especially in the *z*-axis, where only horizontally and coplanarly placed anchors produce unacceptably large errors, and, sometimes, even physically impossible results. To mitigate the problem of the height (*z*-axis) estimation, a good practice is to use an additional sensor and to backup that we used a laser-based ToF altimeter that works very well in indoor environments, but would not work well in outdoor environments, especially during the day, because of the interference caused by the sunlight. The height sensor successfully reduced the *z*-axis error with the RMSE *z* lower than 1 cm, while the error in the *xy*-plane stayed similar, and it is highly advisable to use an appropriate height sensor depending on the usage conditions and requirements.

The PDOP values vary across different anchor configurations and test positions, indicating how geometry affects positioning accuracy, but follow the rule of a smaller expected PDOP value close to the center of the volume. Configuration 1 consistently shows the highest PDOP across all positions, suggesting a poor geometric diversity in anchor placement for this setup. Configuration 4 provides more consistent and usually the lowest PDOP values. It can be observed that across all tested configurations, increasing the height of the UAV’s position reduces PDOP, indicating that a larger 3D spatial separation between anchors and the UAV improves geometry and, consequently, reduces the PDOP. Configurations 2 and 3 produce stable and relatively low PDOP values for all test points. They show a better consistency across varying heights compared to Configuration 1, but with a larger deviation than Configuration 4, especially in some positions close to the edge of the volume stretched by the anchors. These observations suggest that careful 3D placement of anchors will significantly lower the PDOP, thus improving localization accuracy. Configuration 4 stands out as the most geometrically efficient setup among the four and can be taken as a starting point in planning a UWB anchor setup.

UWB positioning technology is based on the principle of the propagation of electromagnetic waves and, therefore, suffers from the propagation phenomena, e.g., reflection, refraction, and attenuation. To properly design a system and reduce positioning errors, it would be good if all the surrounding material properties are considered. This somewhat suggests that there is no straight-forward answer when it comes to setting up the anchor positions, except that it is necessary to provide a good spatial diversity, so that the intersections of direct rays create angles as close to 90° as possible for most potential positions. In addition to the UWB technology, as some recent studies show, it would be possible to use a different communication technology, e.g., Wi-Fi [39] or Bluetooth [40], but with somewhat lower achievable accuracy than the UWB-based positioning.

In dynamic scenarios, where one or more UAVs are moving simultaneously, it is expected that the error in the position estimation will increase, due to the properties of a flight controller, responsible for stabilization, and because of a non-linear sensor fusion. For this technology to be used in swarms of UAVs, it would be necessary to ensure a spatial separation between individual UAVs to be roughly double the largest RMSE value.

## 6. Conclusions

In this paper, we examined the influence of anchor positions on the localization accuracy of UWB-based positioning. The total of four configurations with four to six UWB transceivers was used and all the measurements were made with and without the use of a laser-based ToF altimeter, to provide better results of the altitude estimation. The measurement results proved that the anchor setup is very important and depending on the use case and the space that needs to be covered by the localization systems, the position of anchors needs to offer as much spatial diversity as possible to overcome a poor PDOP. Achieving a good PDOP is mostly the result of a good spatial distribution of anchors and if the positions are chosen correctly, the expected values of the PDOP are below 1.5. Mostly because of the nature of the space where the UWB-based localization is intended, the altitude estimation usually suffers, because of more anchors in the *xy*-plane and therefore a more precise horizontal 2D estimation, and because the overhead anchor at a high altitude is sometimes impossible to place. To overcome that potential problem, it is advised to use an independent source of information in the form of a laser- or ultrasonic-based altitude sensor. The experiments showed that the currently available UWB-positioning technology can provide a positioning accuracy of under 18 cm in all axes of a 3D coordinate system, stretching the uncertainty volume at around 6 dm^3^, which might not be suitable for high-precision applications. With the usage of an additional altimeter, given that it will be able to work in specific conditions and up to certain altitudes, the localization accuracy is almost reduced to two dimensions. Electromagnetically reflexive surfaces or increase in the dimension of the area in which the UWB positioning technology is used would introduce a multi-path propagation. A potential signal loss due to the low power of the transceivers and the low antenna gain, as well as the necessity to decrease the frequency of position updates, would further result in larger positioning errors and create a system with poorer dynamics. Our future work in this field will be towards space and noise modelling to overcome reflection-based errors, finding a solution to reduce the systematic bias in position estimation, and experimenting with navigation in complex spaces with a larger number of anchors.

## Figures and Tables

**Table 1:** Four configurations of UWB anchors were used in the experiments.

Feature	Configuration 1	Configuration 2	Configuration 3	Configuration 4
Number of anchors	4	5	6	6
Layout of anchors	Same horizontal plane at 40 cm height, 2D rectangle covering the area of 4 m × 3 m	Same as Configuration 1, with one elevated central anchor	Same as Configuration 1, with two elevated central anchors	Three anchors at 40 cm, and three anchors elevated to 2 m
Vertical diversity	None	Medium	High	High
Additional anchor(s)	None	One at (1.5, 2, 2) m	Two at (1, 1, 2) m and (1, 3, 2) m	Two symmetrical triangles
Purpose	Baseline setup, minimal number of anchors required for (2)3D trilateration	Test on how a minimal 3D geometry affects vertical accuracy	Improving 3D trilateration by adding an additional elevated anchor	Maximizing spatial coverage and minimizing geometric distortion
Expected effects	Poor *z*-axis accuracy due to coplanar geometry (high *VDOP*)	Improvement in *z*-axis accuracy, similar horizontal accuracy to Configuration 1	Further improvement in *z*-axis accuracy and reduced RMSE overall	Best overall accuracy

**Table 2:** Average RMSE and STDEV in centimeters for all three test points and three different heights. The RMSE and the standard deviation for *z*-axis are shown for cases without and with the usage of a laser altimeter.

	RMSE *x*, [cm]	RMSE *y*, [cm]	RMSE *z*, [cm]	RMSE *z* Altimeter, [cm]	STDEV *x*, [cm]	STDEV *y*, [cm]	STDEV *z*, [cm]	STDEV *z* Altimeter, [cm]
Conf. 1	17.50	17.13	116.04	0.63	2.82	2.06	17.35	0.19
Conf. 2	13.20	13.89	13.67	0.75	3.05	2.22	3.53	0.14
Conf. 3	16.81	13.19	12.86	1.04	4.41	3.70	2.82	0.26
Conf. 4	12.23	15.04	10.64	0.65	1.06	1.06	0.61	0.25

**Table 3:** The values of PDOP in the tested positions for each configuration.

	Configuration 1	Configuration 2	Configuration 3	Configuration 4
P1 H1	10.9248	1.7457	1.3513	1.4204
P1 H2	2.2062	1.7119	1.3910	1.4594
P1 H3	1.6395	1.5346	1.3943	1.3883
P2 H1	13.9789	1.4132	1.3912	1.3339
P2 H2	2.5895	1.3773	1.4840	1.4128
P2 H3	1.7396	1.3443	1.5185	1.4027
P3 H1	11.6819	2.0236	2.0297	1.3601
P3 H2	2.2947	1.8850	1.9123	1.4272
P3 H3	1.6581	1.5703	1.5481	1.5064

## Data Availability

The original contributions presented in this study are included in the article. Further inquiries can be directed to the corresponding author.

## Abbreviations

The following abbreviations are used in this manuscript:

AoA	Angle of arrival
DS-TWR	Double-sided two way-ranging
GNSS	Global Navigation Satellite System(s)
LoS	Line of sight
PDOP	Positional dilution of precision
RGB	Red, Green, Blue (representing the primary colors of the visible spectrum)
RMSE	Root Mean Squared Error
RTK	Real-Time Kinematic
STDEV	Standard deviation
TDoA	Time difference of arrival
ToF	Time of flight
UAV	Unmanned Aerial Vehicle
UWB	Ultra-Wideband
